# Dietary intake and nutritional profiles characterize patients with Inflammatory Bowel Disease from Argentina

**DOI:** 10.3389/fnut.2026.1913858

**Published:** 2026-10-14

**Authors:** Victoria Lambert, Leonela Tantucci, Dafne Cabrera, Marina L. Fernández, Silvia G. Correa, María D. Román, Domingo C. Balderramo, Pablo A. Romagnoli

**Affiliations:** 1 Instituto de Investigaciones en Ciencias de la Salud (INICSA), CONICET-Facultad de Ciencias Médicas, Universidad Nacional de Córdoba, Córdoba, Argentina; 2 Centro de Investigaciones en Nutrición Humana (CENINH), Escuela de Nutrición, Facultad de Ciencias Médicas, Universidad Nacional de Córdoba, Córdoba, Argentina; 3 Fundación Para el Progreso de la Medicina, Córdoba, Argentina; 4 Hospital Privado Universitario de Córdoba, Córdoba, Argentina; 5 Instituto Universitario de Ciencias Biomédicas de Córdoba (IUCBC), Centro de Investigación en Medicina Traslacional "Severo R. Amuchástegui" (CIMETSA) U.A. Consejo Nacional de Investigaciones Científicas y Técnicas (CONICET), Córdoba, Argentina; 6 Centro de Investigaciones en Bioquímica Clínica e Inmunología (CIBICI), Facultad de Ciencias Químicas, Universidad Nacional de Córdoba, Córdoba, Argentina

**Keywords:** Crohn’s disease, dietary fiber, Inflammatory Bowel Disease, nutrition, tryptophan, ulcerative colitis

## Abstract

**Background:**

Diet is increasingly recognized as a modifiable determinant of Inflammatory Bowel Disease (IBD) onset and progression, including patients with Ulcerative Colitis (UC) and Crohn’s Disease (CD); however, comprehensive nutritional characterization of these populations in Latin America remains scarce.

**Objectives:**

To evaluate dietary intake and nutritional profiles among healthy individuals and IBD patients, focusing on fiber intake and the food-source distribution of dietary tryptophan, and to assess differences according to disease phenotype and activity.

**Methods:**

An observational cross-sectional study recruited 14 healthy individuals, 15 UC patients, and 10 CD patients at the Hospital Privado Universitario de Córdoba, Argentina. Dietary intake was assessed using a prospective seven-day food record analyzed with Nutrio V2.1 software and energy-adjusted by the Willett residual method. Plasma tryptophan was measured by validated HPLC and disease activity by the Mayo score (UC) or the Harvey-Bradshaw index (CD).

**Results:**

No significant differences were observed in total energy, macronutrient, or tryptophan intake between groups. In univariate exploratory analyses, weekly consumption frequency was lower than in healthy individuals for cheese in both UC (*p* = 0.017) and CD (*p* = 0.012), and for whole cereals (*p* = 0.010) and hot beverages (*p* = 0.003) in UC. The relative contribution of specific food groups to total dietary tryptophan also differed, being lower for cheese in CD (*p* = 0.037) and for whole cereals in UC (*p* = 0.008), and higher for meat in UC (*p* = 0.031). Dietary fiber intake was lower in UC than in healthy individuals (*p* = 0.033). A negative dietary-plasma tryptophan correlation was identified exclusively in the healthy group (*r* = −0.4681, *p* = 0.0469). None of these differences remained significant after FDR correction for multiple testing. Disease-activity subgroup analyses were based on small sample sizes (*n* as low as 4) and should be interpreted as descriptive observations only.

**Conclusion:**

These hypothesis-generating findings suggest that tryptophan food-source distribution and fiber intake, rather than caloric or macronutrient deficits, may represent candidate nutritional distinctions in IBD patients; however, as none withstood correction for multiple testing, confirmation in larger prospective studies is required before any clinical implications can be drawn.

## 1 Introduction

Inflammatory Bowel Disease (IBD) is a chronic condition characterized by dysregulated immune responses that cause gastrointestinal tract inflammation. Its global prevalence has increased by approximately 47% in recent decades, posing a significant public health challenge (1). The two primary phenotypes, Crohn’s disease (CD) and ulcerative colitis (UC), exhibit distinct clinical presentations and pathophysiological features: CD is characterized by transmural, skip-lesion inflammation that may affect any segment of the gastrointestinal tract, whereas UC involves continuous superficial inflammation restricted to the colon and rectum (2, 3). Despite these topographical differences, both conditions share a complex and incompletely understood pathophysiology, involving alterations in gut microbiota composition (dysbiosis), immune dysregulation, and disrupted intestinal barrier function (4).

The etiology of IBD remains elusive, widely attributed to a multifactorial interplay between genetic susceptibility, environmental and lifestyle factors, dysbiosis, and immune dysregulation (5, 6). Given its chronic, relapsing course, identifying modifiable environmental determinants is essential for refining preventive strategies and clinical management (7). Among these, diet is increasingly recognized as a key factor. Western dietary patterns exert pro-inflammatory effects, whereas Mediterranean-style or prudent diets may confer anti-inflammatory benefits (8–10). However, the causal direction of this relationship remains debated: it is unclear whether unfavorable dietary patterns contribute to disease onset and perpetuation, or whether patients modify their diet reactively in response to symptoms, or both (11, 12). Although nutrient deficiencies and disease-related malnutrition, including sarcopenia (loss of skeletal muscle mass) and micronutrient depletion, are prevalent clinical outcomes post-diagnosis, recent research has increasingly focused on how dietary choices and food group intake fluctuate according to disease activity (e.g., remission vs. active flare-ups), and how these shifts affect nutritional status beyond total energy intake (13, 14).

The interaction between specific nutrients and gut homeostasis is fundamental to IBD pathogenesis. Tryptophan, an essential amino acid, is critical for maintaining intestinal barrier integrity (15). Importantly, beyond total intake, the dietary matrices from which tryptophan is derived—such as dairy products, whole cereals, or meats—are increasingly recognized as significant modulators of its systemic bioavailability, because food source composition and digestibility govern the efficiency of intestinal absorption and subsequent metabolic fate (16, 17). These food sources may differ substantially between patients and healthy individuals, potentially altering the relationship between dietary intake and plasma tryptophan concentrations. Similarly, dietary fiber provides protective effects by enhancing mucosal integrity, modulating the immune response, and restoring microbial balance, thereby improving clinical symptom management and protecting against chronic inflammation and colorectal malignancy (18, 19). Nevertheless, IBD patients frequently restrict fiber intake to alleviate gastrointestinal symptoms (20, 21), and whether such restriction is universally harmful or appropriate in specific clinical contexts—such as in patients with intestinal strictures—remains a subject of ongoing debate (20, 22). Such avoidance nonetheless often results in reduced whole-grain consumption, even when the overall macronutrient distribution remains stable.

Despite growing interest, the extent to which dietary characteristics—specifically fiber intake and the food-source distribution of tryptophan—differ between healthy individuals and IBD patients remains poorly understood, as does the modulation of these patterns by clinical activity and therapeutic management. To address this knowledge gap, we hypothesized that IBD patients would exhibit distinct dietary profiles compared with healthy individuals, particularly regarding fiber intake and the food-source distribution of dietary tryptophan, and that these profiles would differ further between UC and CD phenotypes. This study therefore aimed to evaluate the nutritional characteristics of healthy individuals and patients with IBD, identifying disparities in fiber intake and the relative contribution of various dietary tryptophan sources within a cohort from Argentina. To our knowledge, this is the first simultaneous characterization of these nutritional parameters in an Argentine IBD population, addressing a notable gap in Latin American nutritional research. Our results indicate that nutritional disparities in this population are driven by food group selection rather than caloric deficit, with IBD patients exhibiting reduced fiber intake and a differential distribution of dietary tryptophan sources compared with healthy individuals.

## 2 Materials and methods

### 2.1 Study population

This observational cross-sectional study recruited participants through the Gastroenterology service of the Hospital Privado Universitario de Córdoba (HPUC). All participants were older than 18 years. Inclusion criteria comprised IBD patients with clinical, radiological, and endoscopic diagnosis, and healthy individuals without a history of IBD or active digestive tract pathology diagnosed during the previous 6 months. Exclusion criteria included pregnancy, antibiotic treatment for more than 7 days within the 2 months before inclusion, abdominal surgery within 6 months, and active neoplasia. Healthy comparison participants were recruited from the local community in the same geographic region as IBD patients.

### 2.2 Clinical assessment

Disease extent and behavior in UC and CD patients were recorded following standard anatomical categories (UC: proctitis, left-sided colitis, or extensive colitis; CD: ileal, colonic, or ileocolonic location, with perianal disease recorded as a modifier), consistent with the Montreal classification. Disease activity in patients with IBD was assessed using validated clinical indices: the Mayo score for UC (23) and the Harvey-Bradshaw Index for CD (24).

### 2.3 Dietary assessment

Participants completed a prospective dietary assessment consisting of a seven-day food record, in which all foods and beverages consumed throughout each day were recorded. Prior to data collection, a registered dietitian provided standardized instructions to ensure accurate completion of the records. Reported foods were subsequently classified into predefined food groups according to their nutritional characteristics: fruits; non-starchy vegetables; legumes; whole cereals; refined cereals; bakery product; starchy vegetables; milk and yoghurt; cheeses; meat; eggs; processed meat; seeds and dry fruits; condiments; animal fats; oils; sugary products; sweet beverages; hot beverages (coffee, tea, yerba mate); and alcoholic beverages.

Daily dietary intake, including total energy, macronutrients, dietary tryptophan, and food group consumption, was estimated using Nutrio V2.1 software. To account for total energy intake, dietary consumption variables were adjusted using the residual method proposed by Willett (25).

### 2.4 Sample collection and processing

Blood samples (EDTA plasma) were collected from each participant and coded with alphanumeric identifiers to ensure confidentiality. All samples were stored at −20 °C at the Inflammatory Bowel Disease Laboratory of the Centro de Investigaciones en Medicina Traslacional “Severo Amuchástegui” (CIMETSA) at Instituto Universitario de Ciencias Biomédicas (IUCBC). Plasma tryptophan concentrations were measured using a validated high-performance liquid chromatography (HPLC) method with high sensitivity and specificity, optimized at the Fundación para el Progreso de la Medicina laboratory (26). Triglycerides, total cholesterol, high-density lipoprotein (HDL), and low-density lipoprotein (LDL) were assessed by routine hospital diagnostics at HPUC according to international standards.

### 2.5 Statistical analysis

Statistical analyses were performed using GraphPad Prism (GraphPad Software, San Diego, CA, United States). Data normality was assessed using the Shapiro–Wilk test, complemented by Q-Q plots given its reduced power at small sample sizes. Parametric/non-parametric test selection was confirmed by graphical inspection. Normally distributed variables were compared across groups using one-way ANOVA, followed by Tukey’s *post hoc* test. Non-normally distributed variables were compared between groups using the Kruskal–Wallis test, followed by Dunn’s multiple comparisons *post hoc* test. Associations between dietary and plasma tryptophan levels were evaluated using Spearman’s correlation coefficient. A *p*-value <0.05 was considered statistically significant.

Given the number of comparisons performed, a two-tier approach to multiplicity was adopted. Dietary fiber intake, the food-source distribution of dietary tryptophan, and the dietary–plasma tryptophan correlation were corrected using the Benjamini-Hochberg FDR procedure (q < 0.05) (27). Comparisons stratified by disease activity, individual food-group differences, and lipid parameters were treated as secondary/exploratory given the small resulting subgroups (as low as *n* = 4) and were not corrected.

## 3 Results

### 3.1 Clinical and demographic characteristics of study participants

Table 1 summarizes the main demographic, nutritional and clinical characteristics of the study population. Regarding disease activity, nearly half of patients with UC exhibited mild activity (46.67%), with a slightly smaller proportion in remission (40%), while the CD group was characterized predominantly by remission (60%). In terms of treatment, all UC patients received mesalazine (100%), with azathioprine as a common adjunct (33.3%), whereas CD had a more diverse therapeutic profile, including the use of biological agents such as infliximab, adalimumab, and vedolizumab. Age, sex distribution, and nutritional status category did not differ significantly between the healthy, UC, and CD groups (Kruskal-Wallis test for age, *p* = 0.909; Fisher’s exact test for sex, *p* = 0.638; and Fisher’s exact test for nutritional status, *p* = 0.476).

**Table 1:** Clinical and demographic characteristics of study participants.

Characteristics	Healthy (*n* = 14)	UC (*n* = 15)	CD (*n* = 10)
Age: median (25th, 75th percentile)	39.5 (29, 45)	36 (32, 45)	38.5 (33, 48)
Sex (F/M)	10/4 71.43%/28.57%	8/7 53.33%/46.67%	7/3 70%/30%
Nutritional status			
Underweight	1 (7.14%)	2 (13.33%)	1 (10%)
Normal weight	9 (64.29%)	8 (53.33%)	3 (30%)
Overweight	3 (21.43%)	2 (13.33%)	5 (50%)
Obese	1 (7.14%)	3 (20%)	1 (10%)
Disease activity			
Remission	–	6 (40%)	6 (60%)
Mild activity	–	7 (46.67%)	2 (20%)
Moderate activity	–	2 (13.33%)	2 (20%)
IBD therapy			
*Conventional*			
Azathioprine	–	5 (33.3%)	3 (30%)
Mesalazine	–	15 (100%)	3 (30%)
Corticosteroids	–	3 (20%)	0 (0%)
*Biological*			
Infliximab	–	0 (0%)	1 (10%)
Adalimumab	–	3 (20%)	5 (50%)
Vedolizumab	–	0 (0%)	1 (10%)

A total of 39 individuals were included into three groups: healthy individuals, *n* = 14; Ulcerative colitis group (UC), *n* = 15 and Crohn’s disease group (CD), *n* = 10. Underweight corresponds to BMI lower than 18.5, Normal weight corresponds to a BMI equal or higher than 18.5 or lower than 25, Overweight corresponds to a BMI higher or equal than 25 and lower than 30, and Obese corresponds to a BMI higher than 30.

### 3.2 Differences in the quantity and frequency of food group consumption and nutrient intake

Figure 1 summarizes differences in dietary intake between IBD patients and healthy individuals. Weekly consumption frequency was lower than in healthy individuals for cheese in both UC (*p* = 0.017) and CD (*p* = 0.012), while whole cereals and hot beverages were lower specifically in UC than in healthy individuals (*p* = 0.010 and 0.003, respectively) (Figure 1A). Quantities consumed (g/day) were lower in CD than in healthy individuals for cheese (*p* = 0.045), and lower in UC than in healthy individuals for whole cereals (*p* = 0.010) (Figure 1B). When stratified by disease activity, cheese intake (g/day) was lower in the Active than in the Control CD subgroup (*p* = 0.020) (Figure 1C). In UC patients, whole cereals and fruit intake (g/day) were lower in the Active than in the Control subgroup (*p* = 0.006 and 0.0400, respectively), while eggs and sweet beverages intake were lower in the Active than in the Remission subgroup (*p* = 0.0433 and 0.0354, respectively). No differences were observed in total energy or macronutrient intake between IBD patients and healthy individuals (Table 2), nor across disease activity categories in either CD or UC (Table 3).

**Figure 1:**
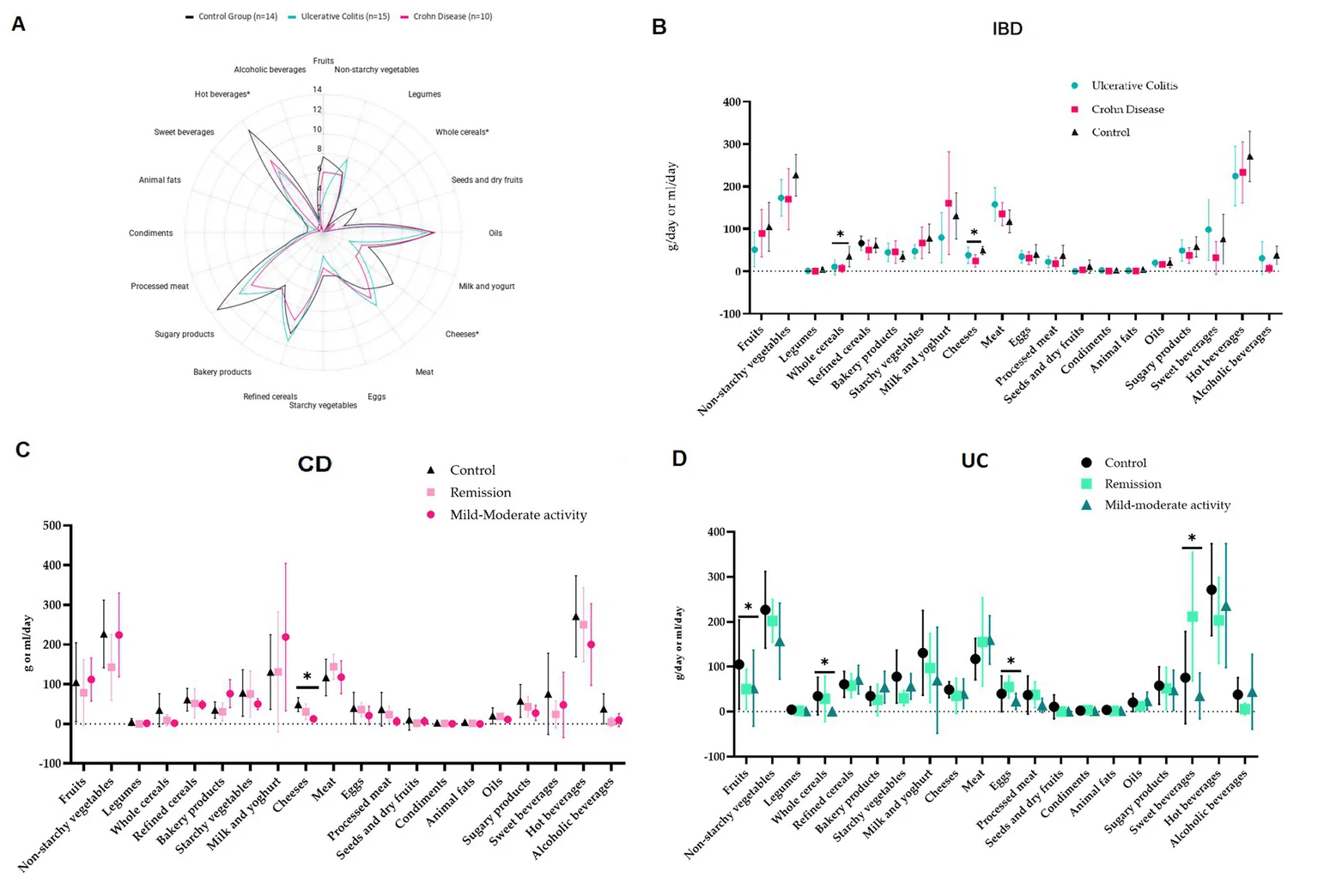
Differences in food group consumption between healthy and IBD groups. Panel **(A)** shows the weekly frequency of food consumption among healthy individuals, ulcerative colitis (UC), and Crohn’s disease (CD) patients. Panel **(B)** presents the daily intake in grams for each food group, comparing healthy individuals, UC, and CD. Panels **(C,D)** display daily intake (g/day) stratified by clinical activity in CD **(C)** and UC **(D)**, respectively. Group sizes: healthy *n* = 14, UC *n* = 15, CD *n* = 10 **(A,B)**; CD Control *n* = 14, Remission *n* = 6, Mild–moderate activity *n* = 4 **(C)**; UC Control *n* = 14, Remission *n* = 6, Mild–moderate activity *n* = 9 **(D)**. Disease-activity-stratified comparisons **(C,D)** involved small subgroups and should be interpreted as exploratory. † *Post hoc* pairwise comparisons (Dunn’s test, Bonferroni-adjusted): Panel **(A)**, cheese, Healthy vs. UC, *p* = 0.017 and Healthy vs. CD, *p* = 0.012; whole cereals, Healthy vs. UC, *p* = 0.010; hot beverages, Healthy vs. UC, *p* = 0.003. Panel **(B)**, cheese, Healthy vs. CD, *p* = 0.045; whole cereals, Healthy vs. UC, *p* = 0.010. Panel **(C)**, cheese, Control vs. Active, *p* = 0.020. Panel **(D)**, whole cereals, Control vs. Active, *p* = 0.006; fruit, Active vs. Control, *p* = 0.040; eggs, Active vs. Remission, *p* = 0.043; sweet beverages, Active vs. Remission, *p* = 0.035.

**Table 2:** Daily energy, macronutrient (protein, carbohydrate, fat), dietary and plasma tryptophan, dietary fiber, and plasma lipid (triglycerides, total cholesterol, HDL, LDL) values in healthy individuals and IBD patients per condition.

Variable	Healthy (*n* = 14)	UC (*n* = 15)	CD (*n* = 10)	*p*-value
Total energy intake, kcal/day	1944.8 ± 505.4	1672.1 ± 442.8	1538.6 ± 318.5	0.090
Total protein intake, g/day	61.4 [58.7–66.9]	64.8 [62.9–78.9]	61.4 [60.2–71.0]	0.380
Total carbohydrate intake, g/day	188.1 ± 57.3	151.5 ± 57.6	140.4 ± 44.3	0.093
Total fat intake, g/day	98.6 ± 24.4	87.5 ± 23.1	82.0 ± 22.1	0.229
Dietary tryptophan intake, mg/day	755.7 [698.5–826.0]	832.0 [738.6–870.2]	819.6 [791.9–864.3]	0.203
Plasma tryptophan, g/dL	0.0115 ± 0.0019	0.0111 ± 0.0017	0.0122 ± 0.0029	0.454
Dietary fiber intake, g/day	**12.8 [8.76–16.2]**	**7.11 [5.51–9.78]**	8.60 [5.51–12.8]	**0.033†**
Plasma triglycerides, mg/dL	111.0 [73.6–132.0]	79.1 [63.4–106.8]	105.5 [94.1–122.8]	0.274
Total cholesterol, mg/dL	173.1 ± 40.2	163.0 ± 23.6	187.7 ± 48.5	0.407
HDL cholesterol, mg/dL	46.4 ± 15.8	41.0 ± 9.62	43.1 ± 12.3	0.595
LDL cholesterol, mg/dL	94.6 ± 23.1	88.0 ± 22.0	104.1 ± 25.9	0.390

Values are presented as mean ± SD for parametric variables and median [IQR] for non-parametric variables. One-way ANOVA/Tukey’s HSD was applied to: total energy, carbohydrate, and fat intake; plasma tryptophan; total, HDL, and LDL cholesterol. Kruskal-Wallis/Dunn (Bonferroni-adjusted) was applied to: total protein and dietary tryptophan intake (energy-adjusted, Willett residual method); dietary fiber intake; plasma triglycerides. *p*-values correspond to the overall three-group comparison, except for † Bolded fiber values differ significantly on post hoc testing (Healthy vs UC, Dunn’s test, Bonferroni-adjusted, *p* = 0.033); the *p*-value shown is this post hoc value, not the overall three-group comparison.

**Table 3:** The same daily energy, macronutrient, plasma tryptophan, dietary fiber, and plasma lipid values, stratified by clinical disease activity in UC or CD patients relative to healthy controls.

Variable	Control (*n* = 14)	UC remission (*n* = 6)	UC active (*n* = 9)	*p*-value
Total energy intake, kcal/day	1944.8 ± 505.4	1741.3 ± 610.1	1633.7 ± 357.6	0.319
Total protein intake, g/day	61.4 [58.7–66.9]	71.2 [63.9–79.3]	62.9 [58.3–77.7]	0.234
Total carbohydrate intake, g/day	188.1 ± 57.3	170.0 ± 67.4	141.2 ± 52.7	0.185
Total fat intake, g/day	98.6 ± 24.4	84.8 ± 31.4	89.0 ± 19.2	0.471
Plasma tryptophan, g/dL	0.0115 ± 0.0019	0.0106 ± 0.0015	0.0114 ± 0.0018	0.612
Dietary fiber intake, g/day	12.8 [8.76–16.2]	7.29 [6.89–13.2]	7.04 [4.73–9.67]	0.055
Plasma triglycerides, mg/dL	111.0 [73.6–132.0]	68.9 [63.4–80.4]	90.1 [68.3–116.2]	0.208
Total cholesterol, mg/dL	173.1 ± 40.2	157.0 ± 12.3	166.0 ± 28.0	0.696
HDL cholesterol, mg/dL	46.4 ± 15.8	43.7 ± 14.1	39.7 ± 7.36	0.551
LDL cholesterol, mg/dL	94.6 ± 23.1	84.7 ± 14.5	89.6 ± 25.7	0.736

Variable	Control (*n* = 14)	CD remission (*n* = 6)	CD active (*n* = 4)	*p*-value
Total energy intake, kcal/day	1944.8 ± 505.4	1565.6 ± 334.9	1484.5 ± 344.6	0.134
Total protein intake, g/day	61.4 [58.7–66.9]	66.2 [60.5–71.7]	60.4 [60.0–63.2]	0.548
Total carbohydrate intake, g/day	188.1 ± 57.3	133.0 ± 51.8	155.2 ± 25.2	0.122
Total fat intake, g/day	98.6 ± 24.4	87.7 ± 21.5	70.5 ± 22.4	0.175
Plasma tryptophan, g/dL	0.0115 ± 0.0019	0.0129 ± 0.0021	0.0111 ± 0.0040	0.436
Dietary fiber intake, g/day	12.8 [8.76–16.2]	6.02 [5.25–10.9]	12.8 [10.7–13.3]	0.146
Plasma triglycerides, mg/dL	**107.0 ± 37.5**	**91.3 ± 19.9**	**208.5 ± 113.8**	**0.023/0.021†**
Total cholesterol, mg/dL	173.1 ± 40.2^a^	180.5 ± 42.9^a^	202.0 ± 75.0^a^	0.691
HDL cholesterol, mg/dL	46.4 ± 15.8^a^	44.3 ± 15.2^a^	40.8 ± 6.72^a^	0.878
LDL cholesterol, mg/dL	94.6 ± 23.1^a^	110.5 ± 29.2^a^	91.3 ± 17.9^a^	0.501

Values are presented as mean ± SD for parametric variables and median [IQR] for non-parametric variables. ANOVA/Tukey and Kruskal-Wallis/Dunn (Bonferroni-adjusted) were applied per variable as in Table 2. *p*-values correspond to the overall three-group comparisons, except for † The bolded plasma triglycerides value in the CD block differs significantly on post hoc testing; the *p*-values shown (CD Active vs Control, *p* = 0.023; CD Active vs CD Remission, *p* = 0.021, both Tukey’s HSD) are post hoc values, not the overall three-group comparison.

### 3.3 Tryptophan intake and plasma levels in Inflammatory Bowel Disease

Total dietary tryptophan intake and plasma tryptophan concentrations did not differ between healthy individuals and IBD patients (Table 2), nor according to disease activity in either UC or CD (Table 3). In contrast, the contribution of specific food groups to total tryptophan intake differed significantly between groups (Figure 2). Cheese and whole cereals contributed less to dietary tryptophan than in healthy individuals (CD, *p* = 0.037; UC, *p* = 0.008, respectively), while meat contributed more in UC than in healthy individuals (*p* = 0.031) (Figure 2A). However, when FDR correction was applied, none remained significant (q ≥ 0.05).

**Figure 2:**
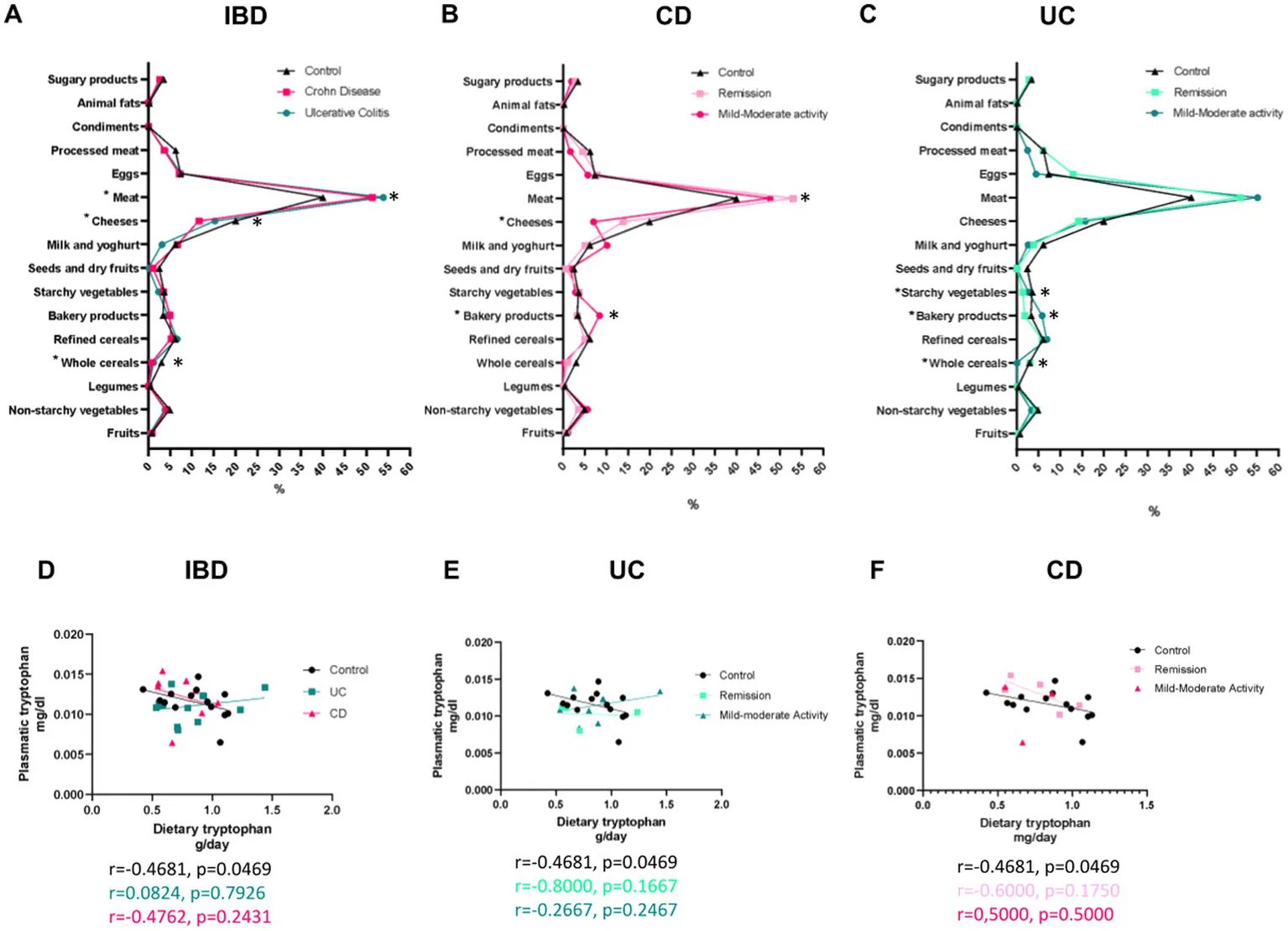
Tryptophan food-source distribution and correlation with plasma tryptophan in Inflammatory Bowel Disease. Panel **(A)** shows the percentage contribution of each food group to total dietary tryptophan intake in healthy individuals, UC, and CD. Panel **(B)** presents the percentage contribution by food group stratified by clinical activity in CD. Panel **(C)** displays the corresponding percentages across activity groups in UC. Panel **(D)** shows the correlation between tryptophan consumed and plasmatic tryptophan between healthy individuals and IBD patients. Panels **(E,F)** show the corresponding correlations stratified by disease activity in UC and CD, respectively. Group sizes: healthy *n* = 14, UC *n* = 15, CD *n* = 10 **(A,D)**; CD Control *n* = 14, Remission *n* = 6, Mild–moderate activity *n* = 4 **(B)**; UC Control *n* = 14, Remission *n* = 6, Mild–moderate activity *n* = 9 **(C)**. The dietary-plasma tryptophan correlation coefficient for the healthy group **(D)** is repeated for reference in Panels **(E,F)** and reflects a single analysis, not repeated testing; none of the correlations shown remained significant after FDR correction (q ≥ 0.05 for all). † *Post hoc* pairwise comparisons (Dunn’s test, Bonferroni-adjusted): Panel **(A)**, meat, Healthy vs. UC, *p* = 0.031; cheese, Healthy vs. CD, *p* = 0.037; whole cereals, Healthy vs. UC, *p* = 0.008. Panel **(B)**, cheese, Control vs. Active, *p* = 0.017; bakery products, Control vs. Active, *p* = 0.033 and Remission vs. Active, *p* = 0.050. Panel **(C)**, whole cereals, Control vs. Active, *p* = 0.005; bakery products, Remission vs. Active, *p* = 0.039; starchy vegetables, Control vs. Remission, *p* = 0.038.

Stratification by disease activity showed that cheese contribution was lower in the Active than in the Control CD subgroup (*p* = 0.017), while bakery products contributed more in the Active than in both the Control (*p* = 0.033) and Remission (*p* = 0.050) CD subgroups (Figure 2B). In UC patients, starchy vegetables and whole cereals contributed less than in the Control subgroup (Remission, *p* = 0.0380; Active, *p* = 0.005, respectively), while bakery products contributed more in the Active than in the Remission subgroup (*p* = 0.0388) (Figure 2C). A negative correlation between dietary and plasma tryptophan levels was identified in the healthy group only (r = −0.4681, *p* = 0.0469), while no significant correlations were observed in either UC or CD patients (Figures 2D–F). This correlation, likewise subjected to FDR correction, did not remain significant (q ≥ 0.05).

### 3.4 Daily fiber intake and plasma lipid profile in Inflammatory Bowel Disease

As shown in Table 2, dietary fiber intake was significantly higher in healthy individuals compared with UC patients specifically (*p* = 0.033); no significant differences were observed between healthy individuals and CD, or between UC and CD subgroups. When stratified by disease activity, fiber intake did not differ across clinical activity categories in either UC or CD (Table 3). After FDR correction, this difference did not remain significant (q = 0.19).

Regarding plasma lipid parameters, triglyceride concentrations varied according to disease activity in CD, with significantly higher values recorded in the mild–moderate activity group compared with both healthy individuals (*p* = 0.0322) and patients in remission (*p* = 0.0230) (Table 3). No significant differences in triglyceride concentrations were observed across disease activity categories in UC. Total cholesterol, HDL, and LDL concentrations were similar across groups and across disease activity categories in both UC and CD.

## 4 Discussion

This study evaluated the dietary and nutritional profiles of IBD patients from Argentina. IBD patients showed a differential food-source distribution of dietary tryptophan relative to healthy individuals, together with lower consumption frequency of cheese in both UC and CD, and of whole cereals and hot beverages specifically in UC, on frequency-based analysis; dietary fiber intake was reduced specifically in UC patients relative to healthy individuals. These differences were not accompanied by changes in total energy or macronutrient intake, suggesting that any nutritional risk in this population may be driven more by food group selection than by caloric deficit. These findings are consistent with observations in other international cohorts, including a Dutch population-based study in which IBD patients had higher animal protein and carbohydrate intakes alongside significantly lower fiber, fruits, and vegetables consumption compared with healthy controls (28). Food group selection also appeared to vary according to disease activity, particularly for cheese intake in CD and several food groups in UC. However, none of these primary comparisons remained significant after correction for multiple testing, and should therefore be considered preliminary. Nevertheless, for several of the activity-stratified comparisons, formal correction could not be meaningfully applied given subgroup sizes as small as *n* = 4, and these are presented descriptively rather than as confirmed findings. These differences appeared to reflect individual dietary choices rather than formal clinical recommendations, and could plausibly relate to specific dietary factors and disease course; however, the cross-sectional design and sample size limit any firm conclusion in this regard.

Notably, the demographic and clinical characteristics of the studied groups (age, sex distribution, nutritional status, disease phenotype/activity, and treatment profile) may also have shaped these dietary comparisons between healthy individuals and IBD patients. Although age, sex distribution, and nutritional status did not differ significantly between groups in the present cohort, both age and sex are recognized, reported modulators of dietary patterns and food group selection in the general population, including in Argentina (29), and their potential influence cannot be fully excluded given the limited sample size.

While total dietary tryptophan intake did not differ between groups, the relative food-source distribution of tryptophan appeared to differ between IBD patients and healthy controls: UC patients derived a greater proportion from meat and a lesser proportion from whole cereals, while CD patients derived a lesser proportion from cheese than healthy controls. Should this pattern be confirmed in larger cohorts, it could carry physiological relevance, as the dietary matrix from which tryptophan is obtained influences its bioavailability and subsequent metabolic fate (16, 17). Systemic free tryptophan levels are physiologically determined by the equilibrium between dietary intake and the activity of diverse metabolic pathways. While a minor fraction of free tryptophan is utilized for protein synthesis and neurotransmitter production, the vast majority of free tryptophan (>95%) is catabolized through the kynurenine pathway, generating metabolites with diverse immunological activities (30). Although alterations in intestinal tryptophan metabolism in IBD are well documented (31), the present data may point to the additional clinical relevance of food-source distribution per se, which varied further according to disease activity in CD, suggesting an interaction between dietary pattern and disease stage that warrants prospective investigation.

The observed shift in food group distribution, comprising a reduction in whole cereals and fiber alongside increased meat consumption, may plausibly have broader implications for the gut-microbiota-immune axis, although this was not directly assessed in the present study. Dietary fiber and whole-grain matrices are essential substrates for the microbial conversion of tryptophan into indole derivatives, which act as ligands for the Aryl Hydrocarbon Receptor (AhR) (32, 33). AhR activation is a recognized regulator of intestinal barrier function and immune homeostasis principally through induction of interleukin-22. We did not measure microbiota composition, indole derivatives, or AhR activation markers in this study; therefore, whether the restrictive dietary patterns identified here curtail production of these protective microbial metabolites and contribute to a pro-inflammatory feedback loop that impedes long-term remission (34, 35) remains a candidate mechanism that warrants direct testing in future mechanistic and metabolomic studies, rather than a conclusion supported by the present data.

Beyond microbial metabolism, tryptophan bioavailability is intrinsically dependent on the nutritional context of its dietary source. Legumes provide a broad array of essential amino acids that are typically limiting in methionine, while cereals, which supply protein predominantly through the germ and bran, are often limiting in tryptophan itself, with overall digestibility ranging between 60 and 86% (36, 37). Complementary mixtures of plant-based proteins can satisfy physiological requirements. However, the restrictive patterns documented in our IBD cohort, driven by perceived symptom risk rather than clinical indication, may compromise this balance and further reduce effective tryptophan bioavailability.

Consistent with current literature, IBD patients frequently report the intentional avoidance of specific food groups, notably dairy and gluten-containing products, due to perceived symptom exacerbation (38). Dairy restriction is of particular concern given its implications for bone health, as dairy restriction may increase the risk of osteoporosis (4). Moreover, while the Mediterranean diet is widely advocated for its anti-inflammatory properties and is increasingly supported by clinical trial evidence in IBD (9), adherence remains low in this population (38, 39), underscoring the gap between dietary recommendations and real-world practice.

Elevated triglyceride concentrations in CD patients with mild-to-moderate disease activity, compared with both healthy individuals and patients in remission, merit particular consideration. However, given the small subgroup sizes inherent to this study, this observation should be interpreted as descriptive rather than confirmatory. This pattern is consistent with prior reports linking active intestinal inflammation to systemic dyslipidaemia via cytokine-mediated suppression of lipoprotein lipase activity, resulting in a characteristic lipid profile with elevated triglycerides and reduced HDL cholesterol (40), though this mechanistic link remains speculative in the present cohort. Interpretation is further complicated by the nutritional status of the CD group: 50% of CD patients in the present cohort were overweight, compared with 21% of healthy controls. Given that elevated BMI is an established, independent driver of hypertriglyceridaemia, adiposity may represent a contributing or confounding factor independently of disease activity, an interpretation consistent with the growing recognition of obesity as an increasingly prevalent comorbidity in IBD, particularly in CD, where more than one-third of patients now meet obesity criteria (12, 41). Disentangling the relative contributions of active inflammation and adiposity to lipid alterations in CD will require multivariable-adjusted analysis in larger, longitudinal cohorts.

The therapeutic heterogeneity within this sample—encompassing mesalazine, azathioprine, corticosteroids, and biologic agents such as infliximab, adalimumab, and vedolizumab—represents an additional source of variability that was not formally controlled for and may have influenced our nutritional findings. Corticosteroids, for example, are well known to increase appetite and alter food choices, while biologic-induced mucosal healing could modify nutrient absorption and gastrointestinal symptoms independently of the clinical activity category assigned. The present sample size precludes formal adjustment for treatment class; disentangling the specific contribution of therapeutic regimen to dietary patterns and nutritional status is therefore an important objective for future, larger studies.

The negative correlation between dietary and plasma tryptophan observed in the healthy group was not observed in UC or CD, and its interpretation should remain guarded: it represents a single, borderline association derived from a small sample rather than firm evidence of a regulatory mechanism. Were it to be replicated in an adequately powered cohort, such a relationship could plausibly reflect a feedback-type interaction between intake and plasma levels, perhaps involving compensatory changes in catabolic pathways such as the kynurenine pathway (42, 43). Whether the absence of a similar relationship in UC and CD patients, even in clinical remission, reflects a genuine disruption of systemic tryptophan homeostasis, or simply reduced power within smaller subgroups, cannot be determined from the present data. Altered intestinal absorption, persistent low-grade mucosal inflammation, and dysbiosis-related changes in microbial tryptophan catabolism remain plausible contributors that warrant direct testing (44). Prospective studies with repeated sampling across disease states and larger sample sizes would be required to determine whether any such disruption precedes, accompanies, or follows inflammatory exacerbations.

This study suggests that, although IBD patients did not differ from healthy participants in systemic nutritional biomarkers or overall macronutrient distribution, disparities may exist in food group selection that merit confirmation in larger, adequately powered studies. This would align with evidence that malnutrition in IBD is an underrecognized condition driven by suboptimal dietary choices and altered nutrient requirements, rather than frank energy insufficiency (20, 45). Importantly, such dietary imbalances would not be expected to be confined to active disease: even during clinical remission, IBD patients have been reported to exhibit reduced adequacy for dietary fiber and key micronutrients compared to healthy individuals (46). This persistent nutritional deficit, would highlight that clinical recovery does not equate to nutritional adequacy, underscoring the potential need for ongoing dietary monitoring to prevent relapse and long-term complications. A specific concern is fiber avoidance: patients with symptomatic intestinal strictures frequently restrict fiber intake to prevent obstructive symptoms, yet this strategy reduces whole-grain consumption and may worsen dysbiosis and precipitate symptomatic flares (18, 39). Given that fiber source and structure are critical determinants of microbial and inflammatory outcomes, self-imposed restriction, when not clinically justified, may ultimately perpetuate rather than attenuate the inflammatory cycle (47).

Personalized nutritional counseling is therefore essential. Practical adaptations, such as modifying food textures (e.g., cooking or processing vegetables to a softer consistency) to permit adequate fiber intake without triggering obstructive symptoms in patients with strictures, represent a tractable clinical strategy (20, 48). An interdisciplinary approach integrating registered dietitians is recommended for optimizing nutritional assessment and supporting long-term remission (49, 50).

The present findings suggest several avenues for future research. Longitudinal studies are warranted to establish whether the shifts in food group distribution observed here, particularly the reduction in whole cereals and fiber alongside the differential sourcing of tryptophan, precede, accompany, or follow changes in disease activity (48). Such designs would also permit assessment of whether targeted reintroduction of specific food groups, rather than broad macronutrient manipulation, translates into measurable improvements in clinical outcomes, an approach increasingly supported by evidence on exclusionary whole-food strategies such as the Crohn’s Disease Exclusion Diet, the Specific Carbohydrate Diet, and the Mediterranean Diet, all of which share an emphasis on the elimination of detrimental components and the incorporation of minimally processed whole foods (48, 51). The differential tryptophan food-source distribution observed between UC and CD phenotypes further suggests that disease-specific dietary approaches may be warranted, rather than a uniform nutritional strategy. Future research should integrate dietary and plasma tryptophan assessments with gut microbiota profiling and inflammatory markers to elucidate the mechanistic pathways by which food-source selection influences intestinal homeostasis (52). Metabolomic characterization of these interactions would be particularly informative and is increasingly advocated by current clinical guidelines as a basis for developing sustainable, phenotype-specific dietary recommendations.

Several limitations must be acknowledged. Most importantly, the small sample size substantially limits statistical power, directly reflected in the fact that none of the dietary fiber intake, tryptophan food-source distribution, or the dietary-plasma tryptophan correlation remained significant after correction for multiple testing. Consequently, all findings reported in this study should be interpreted with considerable caution and regarded as hypothesis-generating rather than confirmatory. Because of this sample size constraint, dietary comparisons could not be stratified or adjusted by age, sex, or nutritional status, and residual confounding by these variables cannot be excluded. Additionally, participants were recruited from a single center in Córdoba, and regional dietary variation across Argentina means these findings should not be generalized to the country as a whole. Larger, multi-center studies are needed to capture regional, age-, sex-, and BMI-related dietary variability in this population. Beyond sample size, the cross-sectional design precludes evaluation of longitudinal changes during disease progression or treatment response. Similarly, although dietary recall bias is inherent in food record methodology, this was mitigated by standardized training of interviewers. A related limitation is that the absence of microbiota or metabolomic data prevents mechanistic inferences regarding the tryptophan pathway. Regarding disease activity, it was assessed using validated clinical indices (Mayo score, Harvey-Bradshaw Index) with inherent subjective components, without corroborating objective biomarkers such as fecal calprotectin or C-reactive protein, limiting assessment of their incremental clinical value. In addition, the considerable heterogeneity in pharmacological treatment across participants was not formally adjusted for and may have influenced dietary and nutritional outcomes independently of disease activity. Furthermore, the absence of multivariable adjustment for BMI in lipid analyses prevents definitive attribution of the observed hypertriglyceridemia in active CD to inflammation versus adiposity. Finally, causal inference is not possible from this design, and the directionality of the associations identified remains to be established prospectively.

## 5 Conclusion

This exploratory study suggests that IBD patients from Argentina may exhibit distinct dietary profiles compared with healthy individuals, including reduced cheese consumption in both UC and CD, reduced whole cereal consumption and lower dietary fiber intake specifically in UC, and a differential food-source distribution of dietary tryptophan, with a greater proportion of tryptophan from meat and a lesser proportion from whole cereals in UC, and a lesser proportion from cheese in CD, than in healthy individuals. These differences were not accompanied by changes in total energy or macronutrient intake, raising the possibility that nutritional disparities in this population may be driven more by food group selection than by caloric deficit. However, none of these differences remained statistically significant after FDR correction for multiple testing, and given the small sample size, they should be regarded as exploratory and hypothesis-generating rather than confirmatory, requiring validation in larger, adequately powered cohorts. Disease activity appeared to further modulate food group choices in both UC and CD phenotypes, though these subgroup analyses were based on very small sample sizes and should be interpreted with particular caution as descriptive observations only. A negative correlation between dietary and plasma tryptophan concentrations was identified in healthy individuals, but not in IBD patients, regardless of clinical stage; whether this reflects a genuine disruption of tryptophan homeostasis in IBD cannot be established from the present data. Overall, these preliminary findings suggest that dietary assessment in IBD may benefit from characterizing food-source distribution in addition to macronutrient quantification, and support further, larger-scale investigation of food group selection, particularly the sources of dietary tryptophan and fiber, according to phenotype and disease activity.

## Data Availability

The de-identified participant data supporting the conclusions of this article will be made available by the authors, upon reasonable request.
